# One-step conversion of tannic acid-modified ZIF-67 into oxygen defect hollow Co_3_O_4_/nitrogen-doped carbon for efficient electrocatalytic oxygen evolution†

**DOI:** 10.1039/d0ra07696a

**Published:** 2020-10-23

**Authors:** Changshui Wang, Jiahui Zhang, Zenong Zhang, Guancheng Ren, Dandan Cai

**Affiliations:** Guangxi Key Laboratory of Low Carbon Energy Materials, School of Chemistry and Pharmaceutical Sciences, Guangxi Normal University Guilin 541004 P. R. China caidandan86@163.com

## Abstract

Controllable structure and defect design are considered as efficient strategies to boost the electrochemical activity and stability of catalysts for the oxygen evolution reaction (OER). Herein, oxygen defect hollow Co_3_O_4_/nitrogen-doped carbon (O_V_-HCo_3_O_4_@NC) composites were successfully synthesized using tannic acid-modified ZIF-67 (TAMZIF-67) as the precursor through a one-step pyrolysis. Tannic acid provides abundant oxygen during the pyrolysis process of the modified ZIF-67, which can contribute to the formation of oxygen defects and the construction of a hollow structure. The existence of oxygen defects is shown by X-ray photoelectron spectroscopy and electron paramagnetic resonance, whereas the hollow structure is confirmed by transmission electron microscopy. The optimized O_V_-HCo_3_O_4_@NC shows good electrocatalytic activity and exhibits a low overpotential of 360 mV at a current density of 10 mA cm^−2^ in 0.1 M KOH due to the hollow structure, abundant oxygen defects, and good electrical conductivity. This work provides valuable insights into the exploration of promising OER electrocatalysts with oxygen defects and special structures.

## Abstract 2

Oxygen defect hollow Co_3_O_4_/nitrogen-doped carbon (O_V_-HCo_3_O_4_@NC) nanocomposites were successfully synthesized by simple one-step pyrolysis of tannic acid-modified ZIF-67 (TAMZIF-67). O_V_-HCo_3_O_4_@NC shows good OER electrocatalytic activity and stability.

## Graphical abstract

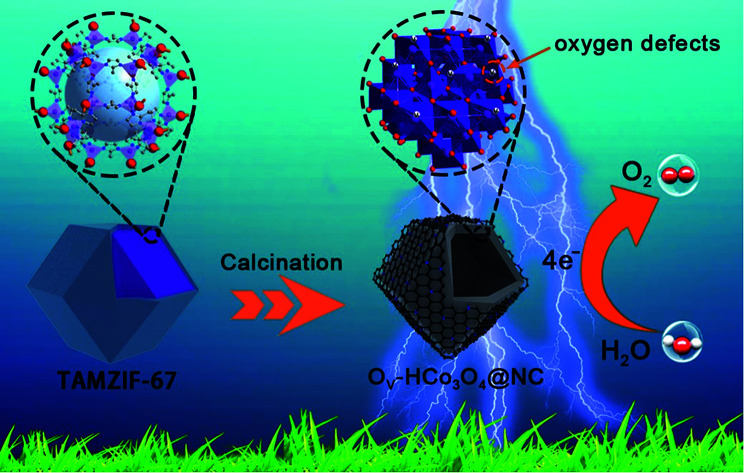

## 1. Introduction

Electrochemical water splitting has been regarded as a promising process for renewable energy high-purity hydrogen evolution.^1^ Unfortunately, the anodic oxygen evolution reaction (OER) limits the cathodic reaction owing to the multistep proton-coupled electron transfer and multiphase reactions.^2^ The development of efficient electrocatalysts is crucial for reducing the electrochemical overpotential and reaction energy barriers. Although some commercial catalysts (RuO_2_ and IrO_2_) show good catalytic activity for the OER, they cannot be used for large-scale application owing to their high cost and low reserves.^3,4^ Therefore, the development of efficient, abundant, and economical electrocatalysts for the OER is one of the main objectives in renewable energy.

To date, non-precious metal electrocatalysts, including transition-metal phosphides, hydroxides, oxides, sulfides, and nitrides, have been developed for the OER.^5–8^ Among the above materials, Co_3_O_4_ has been considered as a promising electrocatalyst due to its high abundance, low cost, and electrochemical stability.^9,10^ Nevertheless, the electrochemical activity is limited due to its poor conductivity and severe nanoparticle aggregation during the OER process.^6,11^ Recently, various strategies have been applied to enhance the OER electrocatalytic performance. On the one hand, the construction of hollow structures and carbon composites can not only provide a large specific surface area to expose more active sites but also enhance the electron conduction and electronic transmission.^3,12^ Benefiting from large pore size, high specific surface area and adjustable composition,^13^ metal–organic frameworks (MOFs) have been considered as promising precursors to prepare hollow structures.^14^ Until now, the hollow structure was prepared though many methods including chemical etching,^15,16^ heat treatment,^17^ self-sacrifice template,^12^ and so on. On the other hand, the introduction of oxygen defects into the structure of oxide can modulate the electron states and surface electronic structure to enhance the electrocatalytic activity and stability for OER. Currently, the main methods for production of oxygen defects are plasma-engraving,^18^ NaBH_4_ treatment,^19^ Ar/air-assisted thermal annealing,^20^ and others. Unfortunately, the above processes are relatively cumbersome, and it is difficult to control recombination in hollow structures and carbon materials during the introduction of oxygen defects. Therefore, a facile synthetic method to synthesize hollow Co_3_O_4_/nitrogen-doped carbon nanocomposites with oxygen defects is urgently needed.

ZIF-67 has been considered as an ideal precursor to prepare Co_3_O_4_ nanomaterials because of its high surface area, redox properties, and uniform dispersion of cobalt centers and dimethylimidazole organic linkers.^21^ However, the as-obtained oxide from ZIF-67 usually requires a two-step pyrolysis and tends to agglomerate due to the absence of oxygen elements and the microporous structure of ZIF-67.^6,22^ To the best of our knowledge, many oxygen atoms exist in tannic acid (TA), and TA has been used as the modifier and a promising oxygen source during ZIF pyrolysis. For example, Liu's group reported that TA molecules were uniformly attached to the surface of ZIF-8 to obtain core–shell composites (ZIF-8@TA) due to the strong adhesion of the catecholic phenolic hydroxyl group.^23^ The surface of the ZIF crystal was attached to TA, which protects the crystal structure from H^+^ etching. Thus, a one-step pyrolysis of TAMZIF-67 could produce an efficient oxide electrocatalyst for OER, but this is still a big challenge.

Herein, the O_V_-HCo_3_O_4_@NC nanocomposites were successfully synthesized by a one-step conversion of TAMZIF-67. The strategies for constructing the hollow structure and introducing the oxygen defects not only provide more active sites but also optimize the electronic structure. The as-optimized nanocomposite is expected to exhibit enhanced electrocatalytic performances for OER.

## 2. Experimental section

### 2.1 Materials

All of the chemicals were of analytical grade purity and were used without further purification. Cobalt nitrate hexahydrate (Co(NO_3_)_2_·6H_2_O), 2-methylimidazole, tannic acid (TA), isopropanol, and KOH were purchased from Aladdin Chemical Reagent Co. Ltd (Shanghai, China). Nafion solution (5 wt%) was purchased from DuPont Co. Ltd (Circleville, OH, USA).

### 2.2 Synthesis of compounds

#### 2.2.1 Synthesis of tannin acid modified ZIF-67 (TAMZIF-67) and ZIF-67

The ZIF-67 sample was synthesized using a facile method. Typically, 2-methylimidazole (9.852 g) and Co(NO_3_)_2_·6H_2_O (0.6072 g) were dissolved in 48 mL of deionized water to form a clear solution. After being stirred for 12 h, the obtained purple product was collected by centrifugation and washed thoroughly with deionized water and methanol three times. Finally, the sample was dried at 80 °C in air for 12 h.

For the preparation of TAMZIF-67, ZIF-67 (0.3 g) and tannic acid (0.5 g) were dispersed in 50 mL of deionized water. The mixed solution was stirred vigorously for 15 min. Then, the TAMZIF-67 was filtered and washed using deionized water and ethanol three times and dried at 80 °C in vacuum for 12 h.

#### 2.2.2 Synthesis of O_V_-HCo_3_O_4_@NC and A-ZIF-67

O_V_-HCo_3_O_4_@NC was obtained by one-step pyrolysis under N_2_ atmosphere. The as-prepared 30 mg of TAMZIF-67 was calcined at 400 °C for 2 h with a slow heating rate of 2 °C min^−1^ to prepare O_V_-HCo_3_O_4_@NC. For comparison, A-ZIF-67 was obtained by a similar method but ZIF-67 was used instead of TAMZIF-67.

### 2.3 Physicochemical characterization

The structures of as-synthesized materials were measured by X-ray diffraction (XRD, Cu Kα, *λ* = 1.54056 Å) with a Rigaku D/max-IIIA diffractometer at 293 K. The morphology and microstructure of the materials were characterized using a field-emission scanning electron microscope (FESEM, Quanta 200 FEG), and their detailed microstructure was further evaluated by transmission electron microscopy (TEM, Model JEM-2011, JEOL, Japan) with a Rontec EDX system. XPS spectra were obtained using a Thermo Fisher Scientific ESCALAB 250. All of the XPS spectra were calibrated with the C 1s peak at 284.8 eV as the binding energy reference. The electron paramagnetic resonance (EPR) spectra were obtained using a Bruker A300 spectrometer (microwave frequency = 9.74 GHz; modulation amplitude = 2 G; modulation frequency = 50 KHz; time constant = 10 ms; conversion time = 25 ms). The Fourier transform infrared spectroscopy (FTIR) spectra were collected using KBr as the reference sample on a Spectrum Two FTIR spectrophotometer (PerkinElmer, Waltham, USA). The TG spectra were measured using a Labsys evo TG-DTA/DSC (Setram, Lyon, France). The Raman spectra were obtained using a Raman spectrometer (Renishaw, London, UK). N_2_ adsorption–desorption isotherms were performed on an A Micromeritics ASAP 2020 analyzer (Micromeritics, Georgia, USA) at liquid nitrogen temperature (77 K).

### 2.4 Electrochemical measurements

All of the electrochemical measurements were carried out in a standard three-electrode system at room temperature using an electrochemical analyzer (760E CH Instrument, purchased from Shanghai Chenhua Instrument Co. Ltd). The glassy carbon electrode (GCE, 3.0 mm in diameter)-modified catalysts served as the working electrode. The counter electrode and reference electrode were a platinum wire and an Ag/AgCl electrode (0.1989 V *vs.* RHE) with saturated KCl filling solution, respectively. In a typical preparation of the catalyst ink, 2.0 mg of catalysts (O_V_-HCo_3_O_4_@NC, A-ZIF-67, and RuO_2_) was dispersed in a mixed solution of 125 μL isopropanol, 125 μL water, and 25 μL 5 wt% Nafion solution. The catalyst ink was obtained by sonication for 30 minutes. Then 2 μL of catalyst ink was pipetted onto the glassy carbon surface and dried in the ambient environment, yielding a catalyst loading of ∼0.20 mg cm^−2^. Prior to the tests, the GCE was polished with a polishing cloth with Al_2_O_3_ powders of different grain sizes (1–0.05 μm) and successively cleaned by ultrasonication in Millipore water, ethanol and deionized water for 10 minutes. In this work, the measured potentials were converted to reversible hydrogen electrode (RHE) with the Nernst equation: *E*_*vs.* RHE_ = *E*_*vs.* Ag/AgCl_ + 0.059 × pH + 0.1989 V. The Tafel slope was calculated according to the Tafel equation: *η* = *b* log *j* + *a*, where *η* is the overpotential, *b* is the Tafel slope, and *j* is the current density.

The linear sweep voltammograms (LSV) were measured at 5 mV s^−1^ in 0.1 M potassium hydroxide (KOH). All of the polarization curves were corrected with 95% *iR* compensation. Electrochemical impedance spectroscopy (EIS) measurements were performed in a 0.1 M KOH solution with an AC amplitude of 5 mV. The electrochemical double layer capacitance (*C*_dl_) values were calculated with the Cyclic voltammetry (CV) curves of O_V_-HCo_3_O_4_@NC and A-ZIF-67 from 0.88 to 0.98 V *versus* RHE at various scan rates of 40, 60, 80, 100, 120, and 140 mV s^−1^. Chronopotentiometry, used to evaluate the stability of O_V_-HCo_3_O_4_@NC and A-ZIF-67, was recorded at a constant current density of 10.0 mA cm^−2^.

## 3. Results and discussion

### 3.1 Compositional and structural characterization of the as-prepared catalysts

**Fig. 1:**
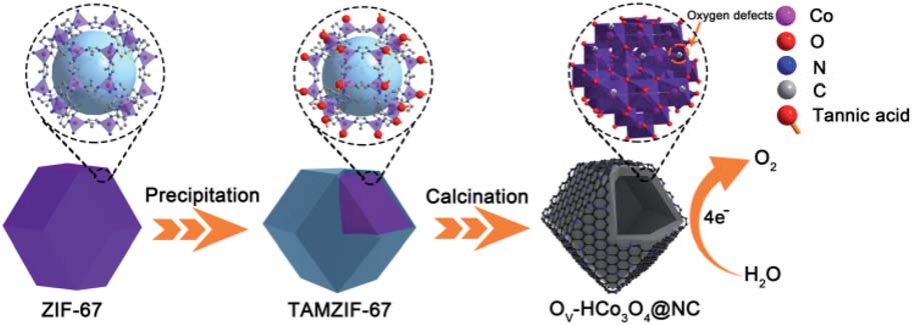
Schematic illustration of the preparation of O_V_-HCo_3_O_4_@NC.

A schematic illustration of the preparation of O_V_-HCo_3_O_4_@NC is presented in Fig. 1. Uniform ZIF-67 nanocrystals were prepared by stirring the mixed solution of 2-methylimidazole and Co(NO_3_)_2_·6H_2_O for 12 h. Then, TAMZIF-67 was synthesized *via* a surface functionalization-assisted modifying process using TA under ambient conditions. During the synthetic process, TA was an effective modifier that created voids in the MOFs with simultaneous surface modification due to its ability to coordinate with metal ions and weak acidity. Finally, O_V_-HCo_3_O_4_@NC was successfully obtained by a simple one-step pyrolysis.

**Fig. 2:**
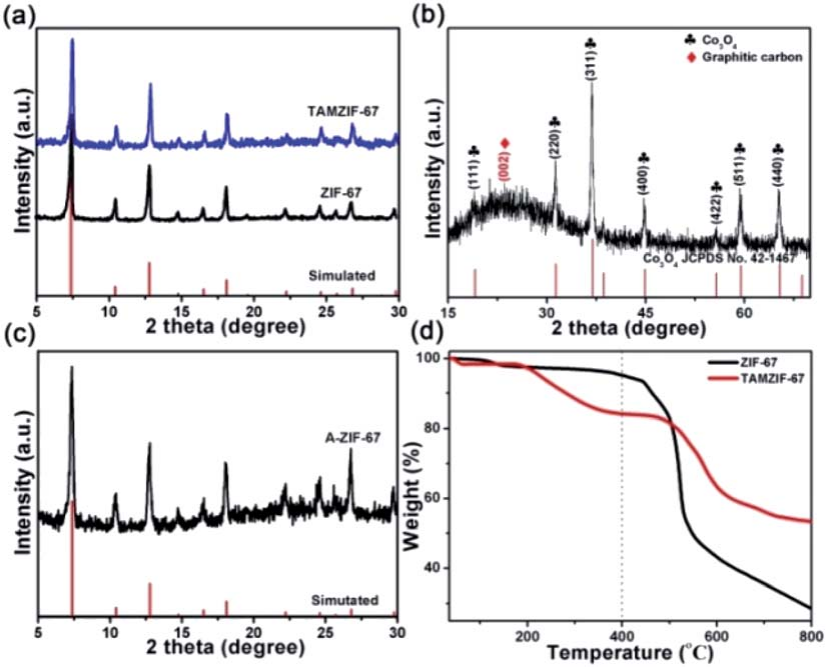
XRD patterns of (a) ZIF-67 and TAMZIF-67, (b) O_V_-HCo_3_O_4_@NC, and (c) A-ZIF-67, (d) TG analysis of ZIF-67 and TAMZIF-67 under N_2_ atmosphere.

X-ray diffraction (XRD) was carried out to investigate the crystal structure of the as-synthesized materials. As shown in Fig. 2a, the obtained ZIF-67 and TAMZIF-67 exhibit the same characteristic diffraction peaks that have been reported for ZIF-67 in previous studies,^7^ which imply that the crystal structure of ZIF-67 was not destroyed. Besides, the characteristic diffraction peaks of O_V_-HCo_3_O_4_@NC are shown in Fig. 2b. Seven prominent and sharp diffraction peaks at 19.1°, 31.2°, 36.7°, 44.8°, 55.6°, 59.2°, and 65.1° can be assigned to the (111), (220), (311), (400), (422), (511), and (440) crystal planes of Co_3_O_4_ (JCPDS file no. 42-1467).^6,24,25^ It should pointed out that the diffraction peak located at 26° can be attributed to the (002) plane of graphitic carbon. For comparison, the structure of ZIF-67 without tannic acid modifying was obtained under similar pyrolysis conditions. As depicted in Fig. 2c, the characteristic diffraction peaks of A-ZIF-67 retain those of ZIF-67. To better study the thermal analysis behavior of ZIF-67 and TAMZIF-67, ZIF-67 and TAMZIF-67 were analyzed using Thermogravimetric Analysis (TG) from 35 °C to 800 °C under N_2_ atmosphere. As shown in Fig. 2d, guest molecules in ZIF-67 and TAMZIF-67 were removed from 35 °C to 200 °C. When the temperature was raised to 400 °C, more TAMZIF-67 mass was lost compared with ZIF-67, indicating that ZIF-67 was easily oxidized by TA.

The functional groups in TAMZIF-67, ZIF-67 and TA were characterized by FTIR spectroscopy. As shown in Fig. S1,† the absorption peaks at 1574 and 761 cm^−1^ are attributed to the stretching vibration and bending mode of the C=N bond in 2-methylimidazole.^26^ The absorption peaks in the region between 900 and 1350 cm^−1^ correspond to the skeletal vibration of the imidazole ring.^27^ The absorption peak at 3400 cm^−1^ comes from the O–H bond of TA.^26^ The characteristic absorption peaks at 1720 and 422 cm^−1^ are assigned to C=O and Co–N bonds,^26–28^ indicating that the main frame of ZIF-67 still exists. To better determine the carbon content of O_V_-HCo_3_O_4_@NC, O_V_-HCo_3_O_4_@NC was evaluated using TG under air. As shown in Fig. S2,† a slight mass loss of 3.34% (*m* (H_2_O)) before 200 °C was observed owing to physically absorbed water molecules on the surface of O_V_-HCo_3_O_4_@NC. The maximum mass decrease (*m* (C), 16.73%) during 200–800 °C was mainly caused by the oxidation of carbon to CO_2_. Therefore, the value (17.31%) of carbon mass content in O_V_-HCo_3_O_4_@NC was calculated according to the following formula (1).1
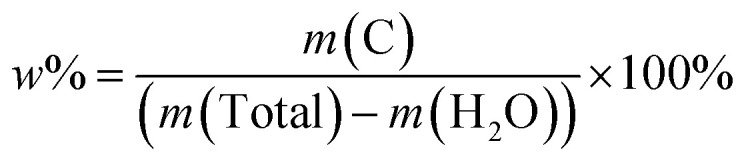


As shown in Fig. S3,† the bands at 193, 478, 517, 617, and 687 cm^−1^ were assigned to the fundamental Raman vibrations of Co_3_O_4_, which is consistent with previous reports.^28,29^ Additionally, the peaks located at about 1335 (D-band) and 1590 cm^−1^ (G-band) are displayed in Fig. S3,† indicating the presence of graphitic carbon in O_V_-HCo_3_O_4_@NC,^15^ which is consistent with the XRD results. To obtain further insights into the porous nature of O_V_-HCo_3_O_4_@NC, the specific surface area and pore size distribution were calculated by Brunauer–Emmett–Teller (BET) and BJH methods, respectively. As shown in Fig. S4,† O_V_-HCo_3_O_4_@NC exhibits a specific surface area of 118.63 m^2^ g^−1^, much higher than that of the Co_3_O_4_/N-PC hybrid (97 m^2^ g^−1^),^30^ Co_3_O_4_ hollow dodecahedra (54.5 m^2^ g^−1^),^31^ and concave-dodecahedron Co_3_O_4_ (16 m^2^ g^−1^).^32^ O_V_-HCo_3_O_4_@NC possesses abundant mesopores with sizes of 4.0 and 6.8 nm. The results demonstrate that the mesoporous structure of O_V_-HCo_3_O_4_@NC should enhance the OER electrocatalytic activity.

**Fig. 3:**
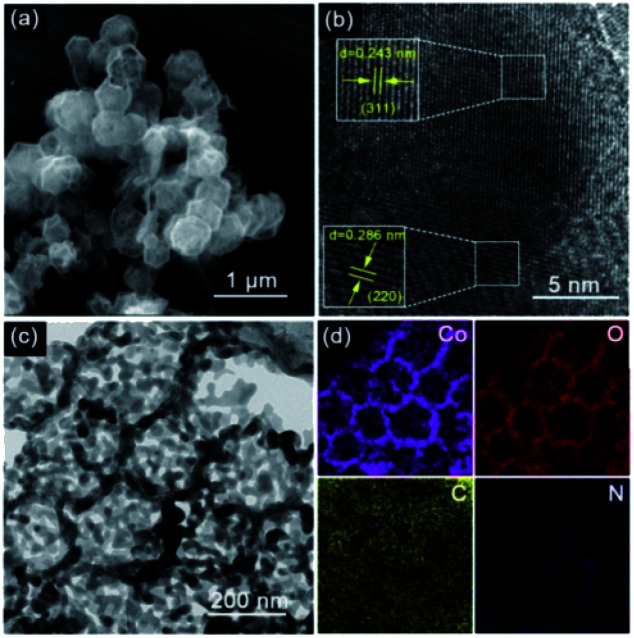
(a) FESEM image, (b) high resolution TEM image, (c) TEM image of O_V_-HCo_3_O_4_@NC, and (d) EDX elemental mapping images of Co, O, C, and N of O_V_-HCo_3_O_4_@NC.

The structural morphology and elemental composition of O_V_-HCo_3_O_4_@NC are shown in Fig. 3. A rhombic dodecahedron shape and hollow structure could be observed for O_V_-HCo_3_O_4_@NC from FESEM image in Fig. 3a. Next, the high-resolution TEM (HRTEM) results confirmed the presence of the Co_3_O_4_ phase (JCPDS no. 42-1467) in Fig. 3b. Two lattice fringes with spacings of 0.286, and 0.243 nm corresponded to the (220), and (311) crystal planes of O_V_-HCo_3_O_4_@NC, respectively, which is consistent with the XRD results. Moreover, as depicted in the TEM image in Fig. 3c, O_V_-HCo_3_O_4_@NC exhibits a hollow structure (about 197 nm in diameter), which was beneficial to the infiltration of the electrolyte and the rapid ions transport, thereby improving its electrocatalytic oxygen evolution activity.^33^ As depicted in Fig. 3d, the elemental mapping demonstrated that C, O, Co, and N elements were uniformly distributed in the O_V_-HCo_3_O_4_@NC. Besides, the morphologies and microstructure of the ZIF-67 and TAMZIF-67 were also characterized by FESEM and TEM. As shown in Fig. S5a,† ZIF-67 nanocrystals exhibit a uniform rhombic dodecahedron shape. The shape of TAMZIF-67 was also a solid rhombic dodecahedron by FESEM and TEM images (Fig. S5b and c†), which revealed that the structure of ZIF-67 was not undermined by TA. Additionally, the shape of A-ZIF-67 maintained the similar morphology of ZIF-67 after pyrolysis at 400 °C (Fig. S5d†).

**Fig. 4:**
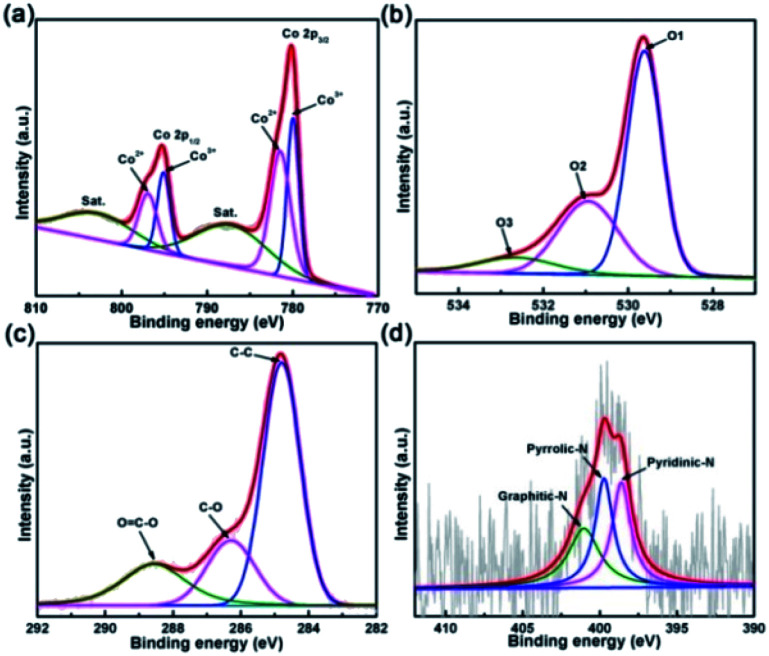
XPS spectra of O_V_-HCo_3_O_4_@NC in the (a) Co 2p, (b) O 1s, (c) C 1s, and (d) N 1s regions.

X-ray photoelectron spectroscopy was used to elucidate the surface chemical composite and bonding states of O_V_-HCo_3_O_4_@NC. As shown in Fig. 4a, the peaks at 780.0 and 795.1 eV correspond to the Co 2p_3/2_ and 2p_1/2_, and the peaks at 781.4 and 796.9 eV are assigned to the Co 2p_3/2_ and 2p_1/2_,^10^ respectively, demonstrating the existence of Co^2+^ and Co^3+^. It is worth noting that low valence Co^2+^ in Co_3_O_4_ with oxygen defects plays a key role in enhancing the OER electrocatalytic activity.^34^ The other two peaks, located at 802.8 and 787.1 eV, belong to shake-up satellites.^4^Fig. 4b shows the three oxygen contributions of O_V_-HCo_3_O_4_@NC. The O1 peak at 529.6 eV is assigned to the typical metal oxygen bond of Co–O.^10^ Meanwhile, the O2 and O3 peaks at 531.0 and 532.6 eV are derived from surface oxygen defect species and absorbed oxygen species.^35,36^ It should be highlighted that oxygen defects are present in O_V_-HCo_3_O_4_@NC. To further verify the existence of oxygen defects, as shown in Fig. S6, *g*-values of O_V_-HCo_3_O_4_@NC were determined by a Bruker EPR spectrometer at room temperature. The *g*-value of 2.006 was assigned to the oxygen defect of Co_3_O_4_ ^10,37^ and other *g*-values of ∼1.98 may originate from metallic Co.^38^ Density-functional theory (DFT) calculations revealed that the introduction of oxygen defects could form the new gap states of Co_3_O_4_, which easily lead to the delocalization of the electrons previously associated with the Co-O bonds.^9,19^ The results may influence the surface electronic structure, improve the electronic conductivity, and thus enhance the electrocatalytic activity for OER.^18,39^ The C 1s high-resolution XPS spectrum is depicted in Fig. 4c, with four main peaks at 288.6, 286.3, and 284.8 eV corresponding to C–O=C, C–O and C–C.^7^ Additionally, the N 1s spectrum (Fig. 4d) shows three strong peaks with binding energy values at 401.0, 399.7, and 398.6 eV,^40^ which correspond to graphitic-N, pyrrolic-N, and pyridinic-N, respectively.

### 3.2 OER performance of the as-prepared catalysts

**Fig. 5:**
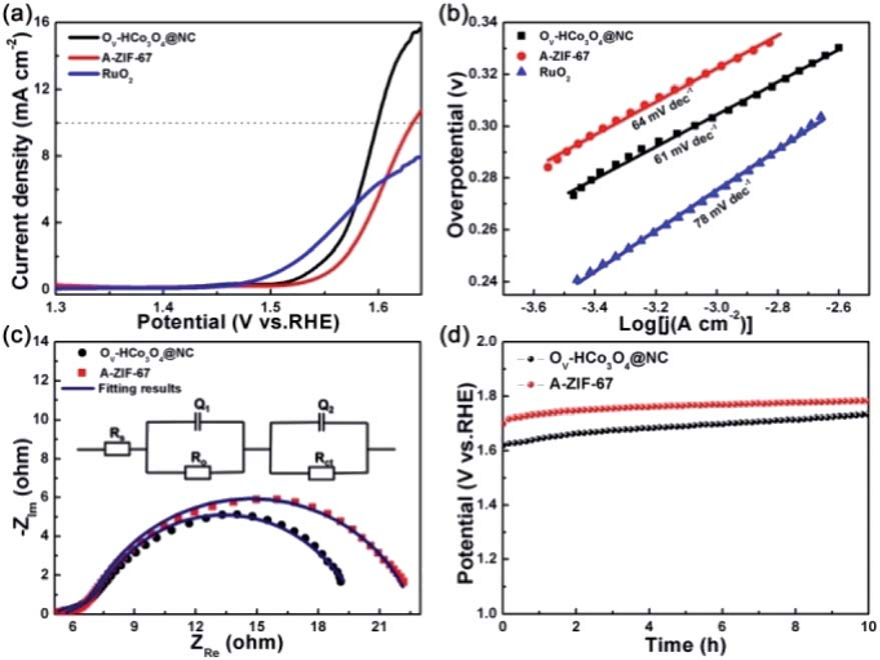
(a) LSV curves of O_V_-HCo_3_O_4_@NC, A-ZIF-67 and RuO_2_. (b) Corresponding Tafel plots. (c) Nyquist plots of O_V_-HCo_3_O_4_@NC and A-ZIF-67 at a potential of 1.57 V *vs.* RHE; the inset is the equivalent electrical circuit used to model the OER kinetics process. (d) Chronopotentiometry measurements for O_V_-HCo_3_O_4_@NC and A-ZIF-67 in OER.

To evaluate the electrocatalytic OER performance of O_V_-HCo_3_O_4_@NC, A-ZIF-67 and RuO_2_, a standard three-electrode system was used in 0.1 M KOH. Polarization curve data were calibrated by *iR* compensation to eliminate the effect of the ohmic potential drop. The LSV curves of O_V_-HCo_3_O_4_@NC, A-ZIF-67, and RuO_2_ are depicted in Fig. 5a. O_V_-HCo_3_O_4_@NC exhibits good OER catalytic activity and requires an overpotential of 360 mV to achieve a current density of 10 mA cm^−2^. In contrast, the overpotential of 400 mV was required for A-ZIF-67 to reach the same current density (10 mA cm^−2^). The results reveal that O_V_-HCo_3_O_4_@NC has a higher OER catalytic activity than A-ZIF-67 and RuO_2_. Compared to previously reported Co_3_O_4_ catalysts, such as RGO/Co_3_O_4_ yolk–shell nanocages (410 mV),^41^ N-doped graphene/Co-embedded porous carbon polyhedron hybrid (430 mV),^42^ and ordered mesoporous Co_3_O_4_ spinels (411 mV),^43^ O_V_-HCo_3_O_4_@NC requires lower overpotential (360 mV) at a current density of 10 mA cm^−2^ in 0.1 M KOH. Meanwhile, the Tafel slopes of O_V_-HCo_3_O_4_@NC, A-ZIF-67 and RuO_2_ were derived from the corresponding LSV curves. As shown in Fig. 5b, O_V_-HCo_3_O_4_@NC has a much smaller Tafel slope (61 mV dec^−1^) than that of A-ZIF-67 (64 mV dec^−1^) and RuO_2_ (78 mV dec^−1^), demonstrating the more favorable OER kinetics of O_V_-HCo_3_O_4_@NC.

EIS was carried out to gain further insight into the interfacial reactions and electrode kinetics in the OER process. The measured impedance spectra data were collected and fitted using the ZSimDemo software. The Nyquist plots and corresponding fitted results are depicted in Fig. 5c. The equivalent circuit composed of *R*_s_, (*Q*_1_, *R*_o_), and (*Q*_2_, R_ct_) is shown in the inset of Fig. 5c. *R*_s_, *R*_o_, and *R*_ct_ represent the solution resistance, oxide film resistance, and the charge transfer resistance at the catalyst/electrolyte interface, respectively. Besides, *Q*_1_ and *Q*_2_ are the constant phase elements (CPE) corresponding to the oxide mass and the interface between the oxide film and the electrolyte, respectively.^28,44^ The value of *R*_s_ (5.5 Ω cm^2^) for O_V_-HCo_3_O_4_@NC is almost consistent with A-ZIF-67 (5.1 Ω cm^2^). Besides, the values of *R*_o_ for O_V_-HCo_3_O_4_@NC and A-ZIF-67 are 0.6 and 0.2 Ω cm^2^, respectively. O_V_-HCo_3_O_4_@NC exhibited a smaller charge transfer resistance (*R*_ct_) of 10.2 Ω cm^2^ than that of A-ZIF-67 (15.8 Ω cm^2^), indicating a faster faradaic process and better OER kinetics.

The electrochemical surface area (ECSA) was calculated to evaluate the specific surface area and the number of catalytically active sites.^45^ The ECSA can be determined using the *C*_dl_.^46^ CV curves were obtained in the non-faradaic region to calculate the *C*_dl_. As shown in Fig. S7,†*C*_dl_ of 51 μF cm^−2^ and 38 μF cm^−2^ were assigned to O_V_-HCo_3_O_4_@NC and A-ZIF-67, respectively. The results imply that O_V_-HCo_3_O_4_@NC has a larger active surface area, which can provide more electrocatalytic active area and facilitate mass transfer. Stability is another important factor in evaluating catalysts in practical applications.^5,47^ As shown in Fig. 5d, the durability of O_V_-HCo_3_O_4_@NC and A-ZIF-67 was tested at a current density of 10 mA cm^−2^ for 10 h. O_V_-HCo_3_O_4_@NC exhibited a lower stable potential (1.61 V *vs.* RHE) than A-ZIF-67 (1.71 V *vs.* RHE), demonstrating the better long-term stability of the O_V_-HCo_3_O_4_@NC catalyst. As depicted in Fig. S8,† O_V_-HCo_3_O_4_@NC remains the hollow structures after OER stability test. Meanwhile, main phase peaks of O_V_-HCo_3_O_4_@NC are well preserved.

## 4. Conclusion

In summary, a facile strategy is proposed to prepare the O_V_-HCo_3_O_4_@NC nanocomposite with hollow structure and oxygen defects by one-step pyrolysis of tannic acid-modified ZIF-67. The as-obtained O_V_-HCo_3_O_4_@NC possesses a hollow structure, abundant oxygen defects, and a combination of nitrogen-doped carbon materials. O_V_-HCo_3_O_4_@NC required an overpotential of 360 mV to reach a current density of 10 mA cm^−2^ in 0.1 M KOH, much lower than that of A-ZIF-67 and RuO_2_ due to its hollow structure, the presence of oxygen defects, and good electrical conductivity. This work will pave the way for developing and designing a high-performance OER electrocatalyst based on modified MOFs.

## Conflicts of interest

There are not conflicts to declare.

## Supplementary Material

RA-010-D0RA07696A-s001
