# Supplementary material for: One-step conversion of tannic acid-modified ZIF-67 into oxygen defect hollow Co3O4/nitrogen-doped carbon for efficient electrocatalytic oxygen evolution
Source: RSC Adv. 2020 Oct 23;10(64):38906–11. doi: 10.1039/d0ra07696a (PMC9057370; doi:10.1039/d0ra07696a)
Supplement: RA-010-D0RA07696A-s001 [file RA-010-D0RA07696A-s001.pdf]

Electronic Supplementary Material (ESI) for RSC Advances.
This journal is © The Royal Society of Chemistry 2020

One-step conversion of tannic acid-modified ZIF-67 into oxygen defects hollow Co_3O_4/nitrogen-doped carbon for efficient electrocatalytic oxygen evolution

Changshui Wang[1], Jiahui Zhang[1], Zenong Zhang, Guancheng Ren, Dandan Cai[*]

Guangxi Key Laboratory of Low Carbon Energy Materials, School of Chemistry and Pharmaceutical Sciences, Guangxi Normal University, Guilin, 541004, P. R. China

Table of Content

**Figure S1**	FTIR spectra of TAMZIF-67, ZIF-67, and TA.
**Figure S2**	TG analysis of O_V-HCo_3O_4@NC under air.
**Figure S3**	Raman spectrum of O_V-HCo_3O_4@NC.
**Figure S4**	(a) N_2 adsorption-desorption isotherms and (b) the pore size distribution of O_V-HCo_3O_4@NC.
**Figure S5**	(a) FESEM image of ZIF-67, (b) FESEM image of TAMZIF-67, (c) TEM image of TAMZIF-67, and (d) FESEM of A-ZIF-67.
**Figure S6**	EPR spectrum of O_V-HCo_3O_4@NC.
**Figure S7**	(a) The double-layer capacitance (C_{dl}) is equivalent to half the slope. Cyclic voltammograms of (b) O_V-HCo_3O_4@NC and (c) A-ZIF-67 in the double layer region at various scan rates.
**Figure S8**	(a) TEM image and (b) XRD pattern of O_V-HCo_3O_4@NC after OER stability test.

*Corresponding author
E-mail: caidandan86@163.com
C. Wang and J. Zhang contributed equally to this work.

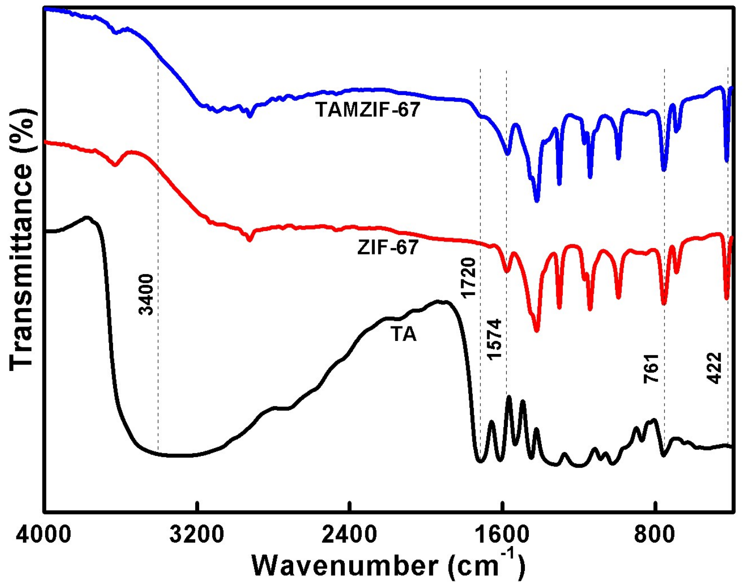

Figure S1 FTIR spectra of TAMZIF-67, ZIF-67, and TA.

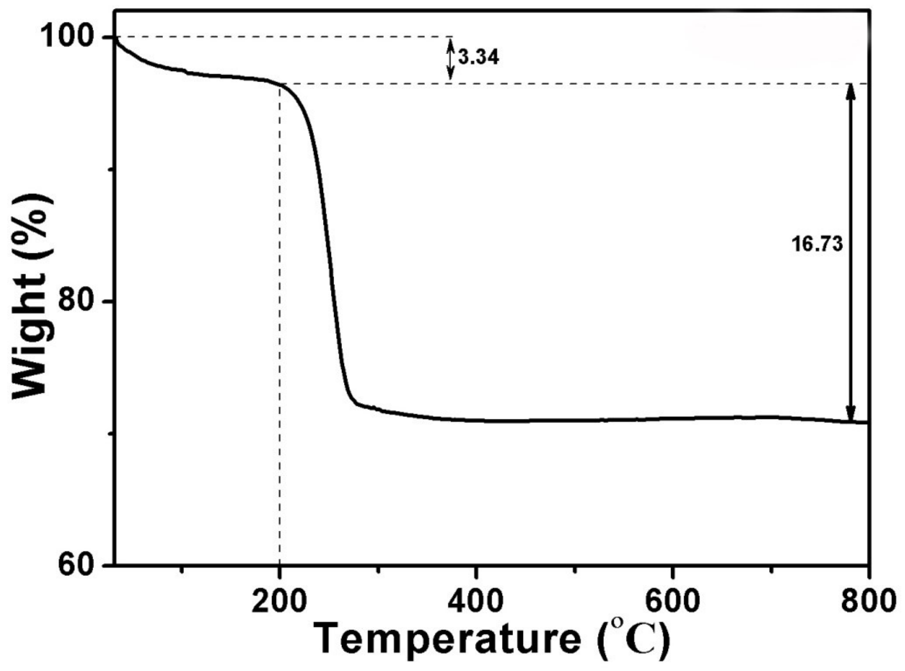

Figure S2 TG analysis of O_V-HCo_3O_4@NC under air.

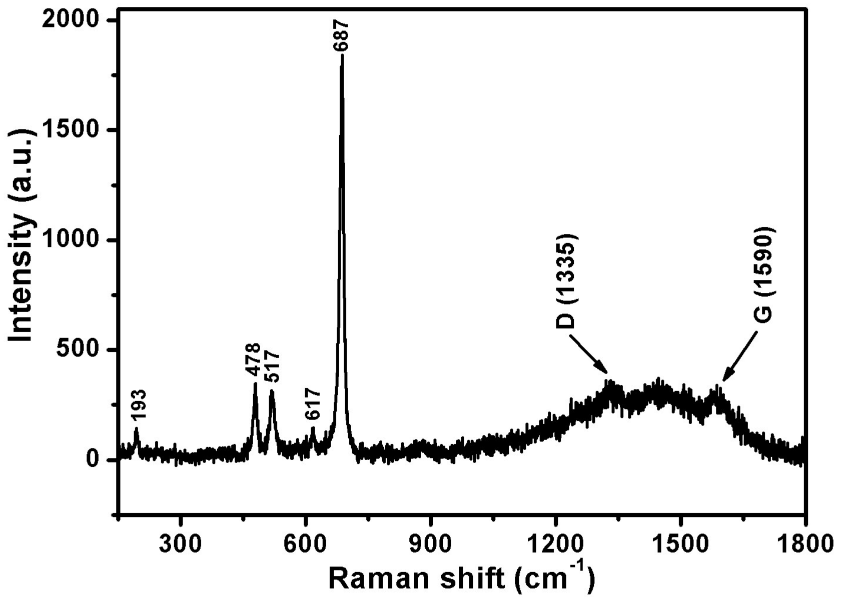

Figure S3 Raman spectrum of O_V-HCo_3O_4@NC.

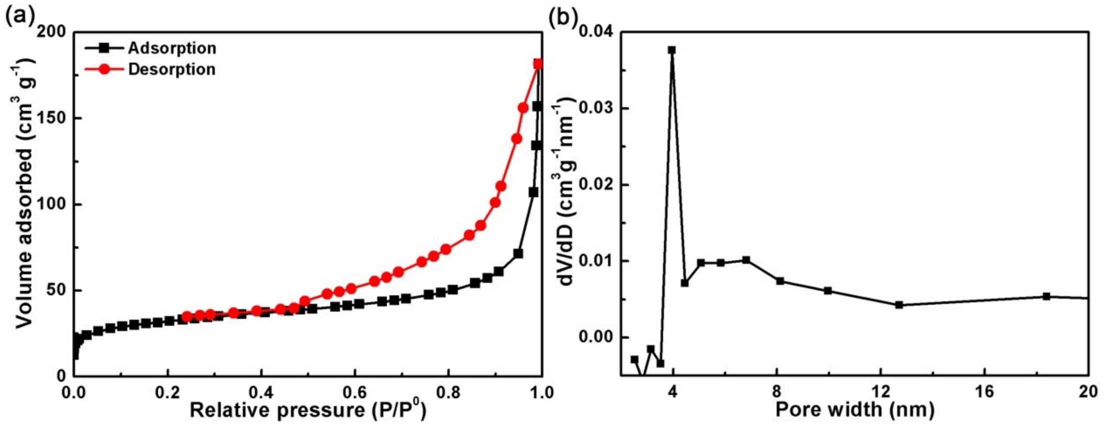

Figure S4 (a) N_2 adsorption-desorption isotherms and (b) the pore size distribution of O_V-HCo_3O_4@NC.

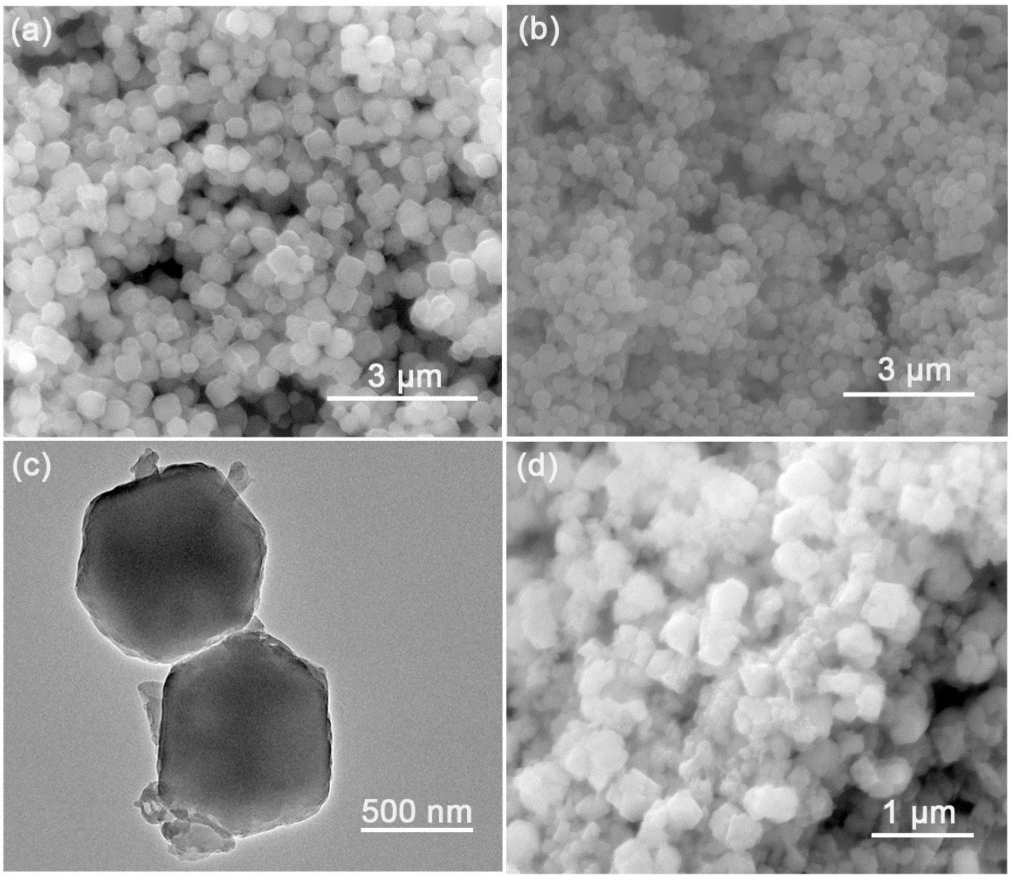

Figure S5 (a) FESEM image of ZIF-67, (b) FESEM image of TAMZIF-67, (c) TEM image of TAMZIF-67, and (d) FESEM of A-ZIF-67.

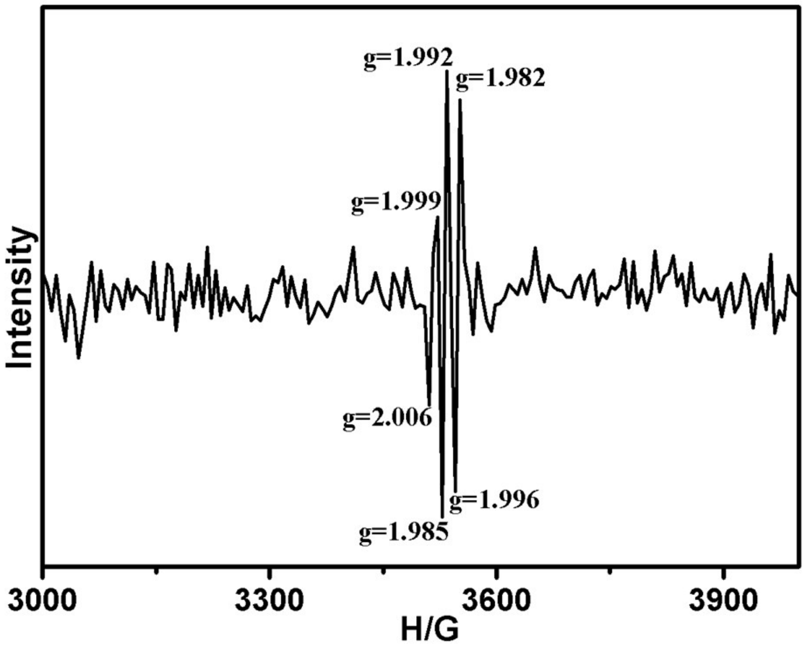

Figure S6 EPR spectrum of O_V-HCo_3O_4@NC.

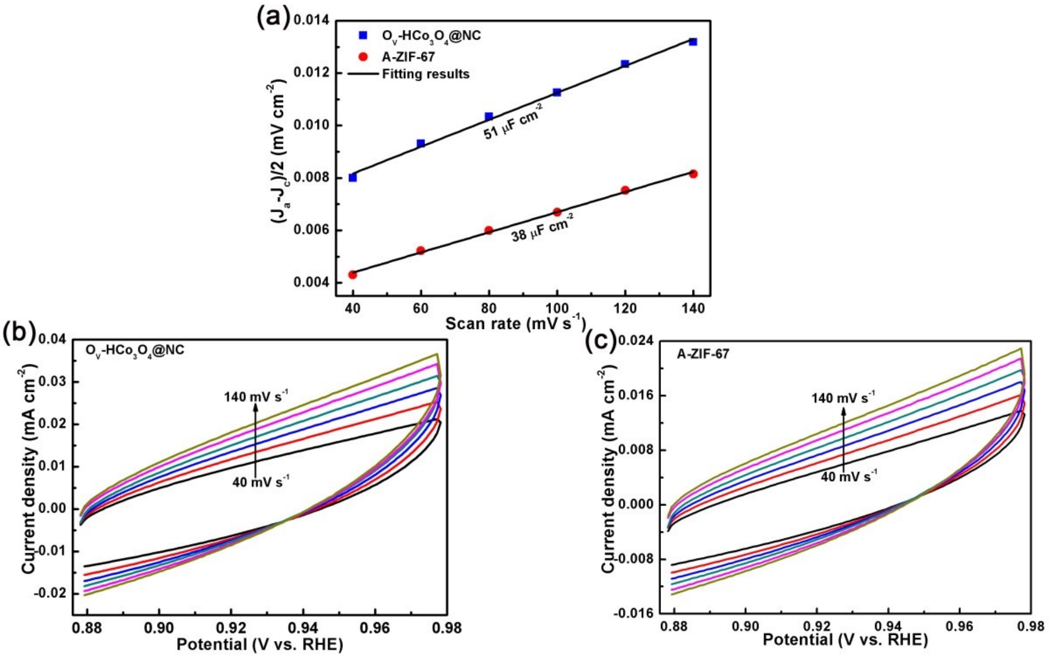

Figure S7 (a) The double-layer capacitance (C_{dl}) is equivalent to half the slope. Cyclic voltammograms of (b) O_V-HCo_3O_4@NC and (c) A-ZIF-67 in the double layer region at various scan rates.

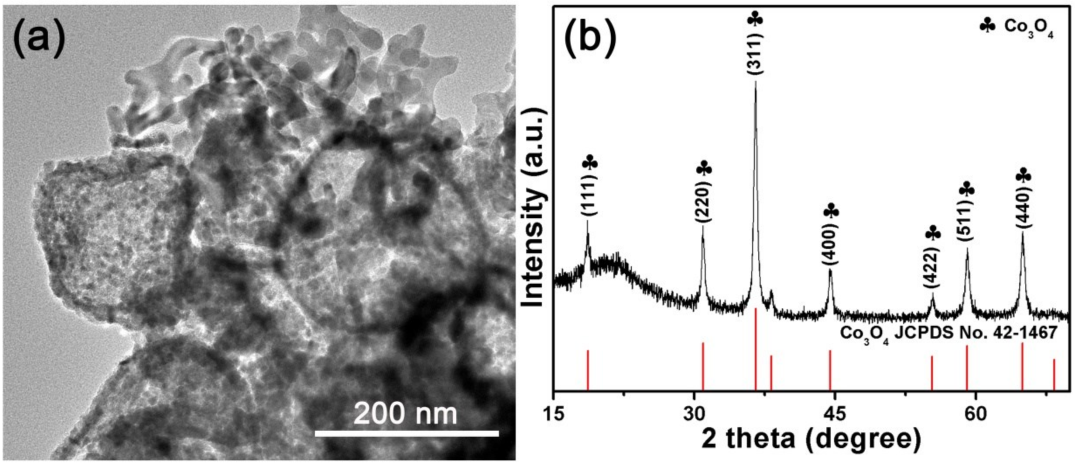

Figure S8 (a) TEM image and (b) XRD pattern of O_V-HCo_3O_4@NC after OER stability test.
